# Optimal assessment of the glomerular filtration rate in older chinese patients using the equations of the Berlin Initiative Study

**DOI:** 10.1007/s40520-023-02657-8

**Published:** 2024-01-31

**Authors:** Yue Yang, Yuan-yuan Jiao, Zheng Zhang, Ding-xin Di, Dan-yang Zhang, Shi-min Jiang, Jia-hui Zhou, Wen-ge Li

**Affiliations:** 1 Department of Nephrology, China-Japan Friendship Hospital, Beijing, China; 2 Department of Nephrology, Fuwai Hospital, Chinese Academy of Medical Science, Beijing, China

**Keywords:** Older, Equation, Chronic kidney disease, Glomerular filtration rate, Chinese

## Abstract

**Aim:**

To evaluate the performances of the various estimated glomerular filtration rate (eGFR) equations of the Chronic Kidney Disease Epidemiology Collaboration, the Berlin Initiative Study (BIS), and the Full Age Spectrum (FAS) in older Chinese.

**Methods:**

This study enrolled Chinese adults aged ≥ 65 years who underwent GFR measurements (via 99Tcm-DTPA renal dynamic imaging) in our hospital from 2011 to 2022. Using the measured glomerular filtration rate (mGFR) as the reference, we derived the bias, precision, accuracy, and consistency of each equation.

**Results:**

We enrolled 519 participants, comprising 155 with mGFR ≥ 60 mL/min/1.73 m^2^ and 364 with mGFR < 60 mL/min/1.73 m^2^. In the total patients, the BIS equation based on creatinine and cystatin C (BIScr-cys) exhibited the lowest bias [median (95% confidence interval): 1.61 (0.77–2.18)], highest precision [interquartile range 11.82 (10.32–13.70)], highest accuracy (P30: 81.12%), and best consistency (95% limit of agreement: 101.5 mL/min/1.73 m^2^). In the mGFR ≥ 60 mL/min/1.73 m^2^ subgroup, the BIScr-cys and FAS equation based on creatinine and cystatin C (FAScr-cys) performed better than the other equations; in the mGFR < 60 mL/min/1.73 m^2^ subgroup, all equations exhibited relatively large deviations from the mGFR. Of all eight equations, the BIScr-cys performed the best.

**Conclusions:**

Although no equation was fully accurate in the mGFR < 60 mL/min/1.73 m^2^ subgroup, the BIScr-cys (of the eight equations) assessed the eGFRs of the entire population best. A new equation is urgently required for older Chinese and even East Asians, especially those with moderate-to-severe renal insufficiency.

**Supplementary Information:**

The online version contains supplementary material available at 10.1007/s40520-023-02657-8.

## Introduction

Life expectancy has increased dramatically over the past decades, attributable to improved living conditions, increased socioeconomic status, and better healthcare, and populations are aging rapidly worldwide [1]. Evaluation of renal function in the older is important for several reasons. First, aging is associated with gradual impairment of the glomerular filtration rate (GFR), which exhibits marked individual variations [2]. Second, an accurate GFR is required when calculating drug doses, especially for drugs eliminated by the kidneys [3]. Third, a low estimated GFR (eGFR) is the main criterion for diagnosis of chronic renal failure, timing of renal replacement therapy, and evaluation for kidney transplantation. Many older patients have underlying diseases, rendering accurate GFR assessment more important but also more difficult [4].

The GFR is the best index of kidney function in both healthy and disease states. However, GFR assessment via clearance of inulin, iohexol, or 125I-iothalamate is invasive, inconvenient, and too costly for use in everyday practice [5]. Renal emission computed tomography (ECT) examination using Technetium-99m-diethylene triamine penta-acetic acid (99mTc-DTPA) as a contrast agent is a method of determining kidney function using radionuclides that was first applied in the 1970s [6]. 99mTc-DTPA renal dynamic imaging [7] has been recommended by the Nephrology Committee of the Society of Nuclear Medicine [8], and is widely used in clinical practice. This radionuclide does not bind to plasma proteins, is only filtered through the glomerulus, is not secreted by the tubules, and is rapidly excreted in the urine, thus accurately reflecting the glomerular filtration capacity. When 99mTc-DTPA enters the human body, it emits γ-rays with certain penetrating power. These photons are detected and recorded by SPECT and processed by a computer to obtain images of organs, tissues or lesions and ultimately information on the morphology, location, size and functional details of the organs.

Several GFR equations based on the serum creatinine (SCr) and/or serum cystatin C (SCys) levels have been developed. Here, we evaluated the Chronic Kidney Disease Epidemiology Collaboration (CKD-EPI) equations [9, 10] used most widely in clinical practice, the Berlin Initiative Study (BIS) equations [11] (which were specially developed for the older), and the Full Age Spectrum (FAS) equations [12, 13] (applicable across the entire age spectrum). We explored which equation best assessed the GFR (i.e., minimal bias and maximal accuracy) in older Chinese.

## Materials and methods

### Study design

This retrospective cohort study included all non-dialysis patients aged ≥ 65 years who underwent GFR measurement (99Tcm-DTPA) between May 2011 and August 2022 in our Hospital. Those with acute kidney failure, on dialysis, who were dehydrated, and who exhibited fluid overload were excluded.

### Data collection and measurements

Clinical information including laboratory data (SCr and SCys levels) and demographic parameters (age, sex, and medical history) were obtained from the electronic medical records. SCr was measured using an enzymatic method after fasting prior to GFR assessment. Weight and height were recorded before GFR measurement. The body mass index (BMI) was the weight (kg) divided by the height squared (m^2^).

The measured GFR (mGFR) was determined using the 99Tcm-DTPA dynamic imaging method. Before imaging, patients fasted for at least 6 h, consumed 150–300 mL water 30 min before assessment, and emptied their bladder. Each individual was placed supine, and 99Tcm-DTPA was administered as a “bolus” (using a SPECT apparatus). The total and lateral renal GFRs were computed by the imaging system, and the results were normalized to a 1.73 m^2^ body surface area (BSA) using the Dubois method [14].

### Estimated GFR equations

The eGFR was calculated using the following equations. The CKD-EPI equations comprises three forms [9, 10]: the SCr-based CKD-EPIcr, SCys-based CKD-EPIcys, and SCr- and SCys-based CKD-EPIcr-cys. The BIS equation has two forms [11]: the SCr-based BIScr and SCr- and SCys-based BIScr-cys. The FAS equation includes three forms [12, 13]: the SCr-based FAScr, SCys-based FAScys, and SCr- and SCys-based FAScr-cys. In addition, as the new European Kidney Function Consortium (EKFC) equation has been modified from the FAScr and is superior to the FAScr [15], we used this equation rather than the FAScr. The equations are shown in Supplementary Table 1.

### Statistical analysis

SPSS ver. 20.0 and MedCalc ver. 20.0.15 were used to perform the statistical analysis. Baseline characteristics are presented as medians (interquartile range) for continuous variables and as numbers or percentages for categorical variables. The performance of each equation in terms of assessing the GFR was evaluated by calculating the bias, precision, and accuracy. Bias was defined as the median difference (MD) between the mGFR and eGFR. Precision was defined as the interquartile range of bias. Accuracy was defined as the proportion of eGFR values within 30% of the mGFR (P30). The KDIGO guidelines state that the P30 should be > 90% [16]. We generated Bland–Altman plots to examine the consistency (precision and mean bias) of the mGFR and eGFR data. The significance level was set to P < 0.05. We analyzed the overall cohort and two subgroups (mGFR < 60 and mGFR ≥ 60 mL/min/1.73 m^2^).

The study was approved by our ethics committee (approval no. 2018–43-K32), and all procedures adhered to the Declaration of Helsinki. All participants provided written informed consent.

## Results

### Patient characteristics

From an initial population of 780 participants, 519 older participants (326 males and 193 females) were selected. In the mGFR ≥ 60 mL/min/1.73 m^2^ (N = 155) subgroup, the median age was 69 years (67–74 years), and 107 patients were male. In the mGFR < 60 mL/min/1.73 m^2^ subgroup (N = 364), the median age was 70 years (67–76 years), and 219 patients were male. The median mGFRs in these two subgroups were 73.42 (67.17–83.59) and 36.87 (23.13–47.35) mL/min/1.73 m^2^, respectively. The SCr and SCys levels, mGFRs, and eGFRs differed between the subgroups (Table 1).

**Table 1:** Clinical characteristics of 519 participants aged 65 years and older

Characteristic	Total	mGFR ≥ 60 ml/min/1.73m2	mGFR < 60 ml/min/1.73m2	P
Participants, n (%)	519 (100)	155 (29.9)	364 (70.1)	
Median age, years	70 (67, 76)	69 (67.74)	70 (67.76)	
Gender (male/female)	326/193	107/48	219/145	
BSA (m^2^)	1.79 (1.65, 1.91)	1.78 (1.67, 1.91)	1.79 (1.64, 1.91)	
SCr (mg/dL)	1.42 (0.96, 2.63)	0.83 (0.70, 1.00)	2.00 (1.36, 3.59)	< 0.001
SCys (mg/L)	1.56 (1.03, 2.55)	0.93 (0.81, 1.03)	2.02 (1.47, 3.31)	< 0.001
mGFR (mL/min/1.73m^2^)	45.00 (29.95, 65.21)	73.42 (67.17, 83.59)	36.87 (23.13, 47.35)	< 0.001
eGFR (mL/min/1.73 m^2^)				
CKD-EPIcr	39.48 (19.00, 67.42)	79.86 (65.21, 92.48)	26.76 (12.69, 42.90)	< 0.001
CKD-EPIcys	40.75 (20.98, 70.18)	80.46 (68.63, 94.53)	28.82 (15.17, 43.91)	< 0.001
CKD-EPIcr-cys	41.13 (19.82, 72.83)	82.10 (73.49, 92.44)	29.05 (15.37, 44.85)	< 0.001
EKFC (modified FAScr)	37.76 (19.12, 62.30)	72.57 (60.75, 83.80)	25.53 (13.33, 41.23)	< 0.001
FAScys	42.29 (25.31, 63.17)	69.72 (62.75, 79.53)	31.97 (19.83, 44.58)	< 0.001
FAScr-cys	39.54 (22.79, 60.54)	68.60 (60.87, 77.53)	29.10 (17.39, 41.73)	< 0.001
BIScr	41.33 (24.39, 60.55)	68.50 (57.92, 81.35)	30.50 (18.79, 44.45)	< 0.001
BIScr-cys	41.77 (24.28, 64.98)	74.14 (65.10, 81.63)	31.59 (20.05, 45.02)	< 0.001

Data are presented as the median (IQR) and n (%)

*BSA* body surface area, *SCr* serum creatinine, *SCys* serum cystatin C, *mGFR* measured glomerular filtration rate, *eGFR* estimated glomerular filtration rate, EKFC: European Kidney Function Consortium, FAS: Full Age Spectrum, *BIS* Berlin Initiative Study, *CKD-EPI* Chronic Kidney Disease Epidemiology, To convert SCr from mg/dl to umol/L, multiply by 88.4

### The bias, precision, and accuracy of the eGFR equations

In general, when the mGFR served as the reference, equations based on SCys or both SCys and SCr were better than equations based only on SCr (Table 2); all eGFRs were relatively low. The BIScr-cys equation was the best of all eight equations. The MD of the eGFR equations ranged from 1.61 to 6.21. The BIScr-cys equation evidenced the least bias [MD (95 CI%): 1.61 (0.77–2.18)], followed by the CKD-EPIcys [1.99 (0.63–3.28)] and CKD-EPIcr-cys [2.33 (1.32–3.36)]. The BIScr-cys also exhibited the highest precision [IQR: 11.82 (10.32–13.70)] followed by the FAScr-cys [11.90 (10.86–13.15)] and BIScr [12.92 (11.30–14.22)]. Regarding accuracy, the P30 was highest for the BIScr-cys Eq. (81.12%) followed by FAScr-cys (75.53%) and FAScys (75.53%).

**Table 2:** Bias, precision and accuracy of different equations in elderly patients according to measured GFR

Pairwise comparisons	Bias	Precision	Accuracy
	MD (95% CI)	IQR (95% CI)	P30 (%)
Whole cohort (N = 519)			
CKD-EPIcr	4.05 (2.88, 5.87)	16.08 (13.93, 18.19)	60.31
CKD-EPIcr-cys	2.33 (1.32, 3.36)	16.22 (14.09, 18.14)	69.56
CKD-EPIcys	1.99 (0.63, 3.28)	16.46 (14.69, 18.89)	68.40
EKFC	6.21 (4.99, 7.04)	13.18 (12.05, 15.18)	63.78
FAScys	6.21 (4.99, 7.04)	14.07 (12.53, 15.75)	75.53
FAScr-cys	4.25 (3.24, 4.97)	11.90 (10.86, 13.15)	75.53
BIScr	3.28 (2.29, 4.36)	12.92 (11.30, 14.22)	74.18
BIScr-cys	1.61 (0.77, 2.18)	11.82 (10.32, 13.70)	81.12
Measured GFR ≥ 60 mL/min/1.73 m^2^(N = 155)			
CKD-EPIcr	−4.00 (−6.92, −1.65)	22.25 (18.26, 26.05)	81.29
CKD-EPIcr-cys	−8.15 (−9.50, −4.29)	17.03 (14.54, 21.18)	87.74
CKD-EPIcys	−5.71 (−8.71, −2.86)	21.87 (16.98, 28.30)	81.29
EKFC	2.43 (0.17, 5.26)	19.80 (17.03, 24.16)	87.10
FAScys	4.92 (2.41, 7.40)	18.69 (14.11, 21.71)	90.97
FAScr-cys	6.47 (3.51, 7.91)	14.50 (11.92, 17.28)	92.90
BIScr	4.48 (2.91, 7.59)	18.07 (15.18, 22.80)	86.45
BIScr-cys	1.46 (−0.56, 3.61)	17.13 (12.72, 18.79)	96.13
Measured GFR < 60 mL/min/1.73 m^2^(N = 364)			
CKD-EPIcr	6.36 (4.94, 7.42)	11.67 (10.46, 13.49)	51.37
CKD-EPIcr-cys	5.10 (3.43, 5.70)	12.13 (10.81, 13.87)	61.81
CKD-EPIcys	3.85 (2.71, 5.19)	13.84 (12.23, 15.77)	62.91
EKFC	6.92 (6.01, 7.77)	11.16 (9.98, 12.93)	53.85
FAScr-cys	3.64 (2.56, 4.62)	10.98 (9.68, 12.05)	68.13
FAScys	0.89 (−0.18, 1.89)	12.57 (10.72, 14.74)	68.96
BIScr	2.89 (1.74, 4.02)	10.74 (9.42, 12.08)	68.96
BIScr-cys	1.63 (0.77, 2.04)	10.56 (9.15, 12.50)	74.73

Data were showed as value (95% confidence interval, 95% CI). CIs for the metrics were calculated by means of bootstrap methods (1000 bootstraps)

*MD* median difference, *IQR* interquartile range, *EKFC* European Kidney Function Consortium, *FAS* Full Age Spectrum, *BIS* Berlin Initiative Study, *CKD-EPI* Chronic Kidney Disease Epidemiology

In the mGFR ≥ 60 mL/min/1.73 m^2^ subgroup, compared with the mGFR, the eGFRs calculated using the CKD-EPI equations were relatively high and those calculated using the BIS and FAS equations relatively low (Table 2). The BIScr-cys equation exhibited the least bias [MD (95 CI%): 1.46 (–0.56, 3.61)] and the best accuracy (P30: 96.13%). The FAScr-cys equation evidenced the highest precision [IQR (95 CI%): 14.50 (11.92–17.28)]. In the mGFR < 60 mL/min/1.73 m^2^ subgroup, compared with the mGFR, all eGFR equations yielded relatively low values. The BIScr-cys equation afforded the highest precision [IQR: 10.56 (9.15–12.50)] and accuracy (P30: 74.73%); the FAScys equation exhibited the lowest bias [MD (95% CI): 0.89 (− 0.18, 1.89)].

### Bland–Altman plots for the eight equations compared with the mGFR

**Fig. 1:**
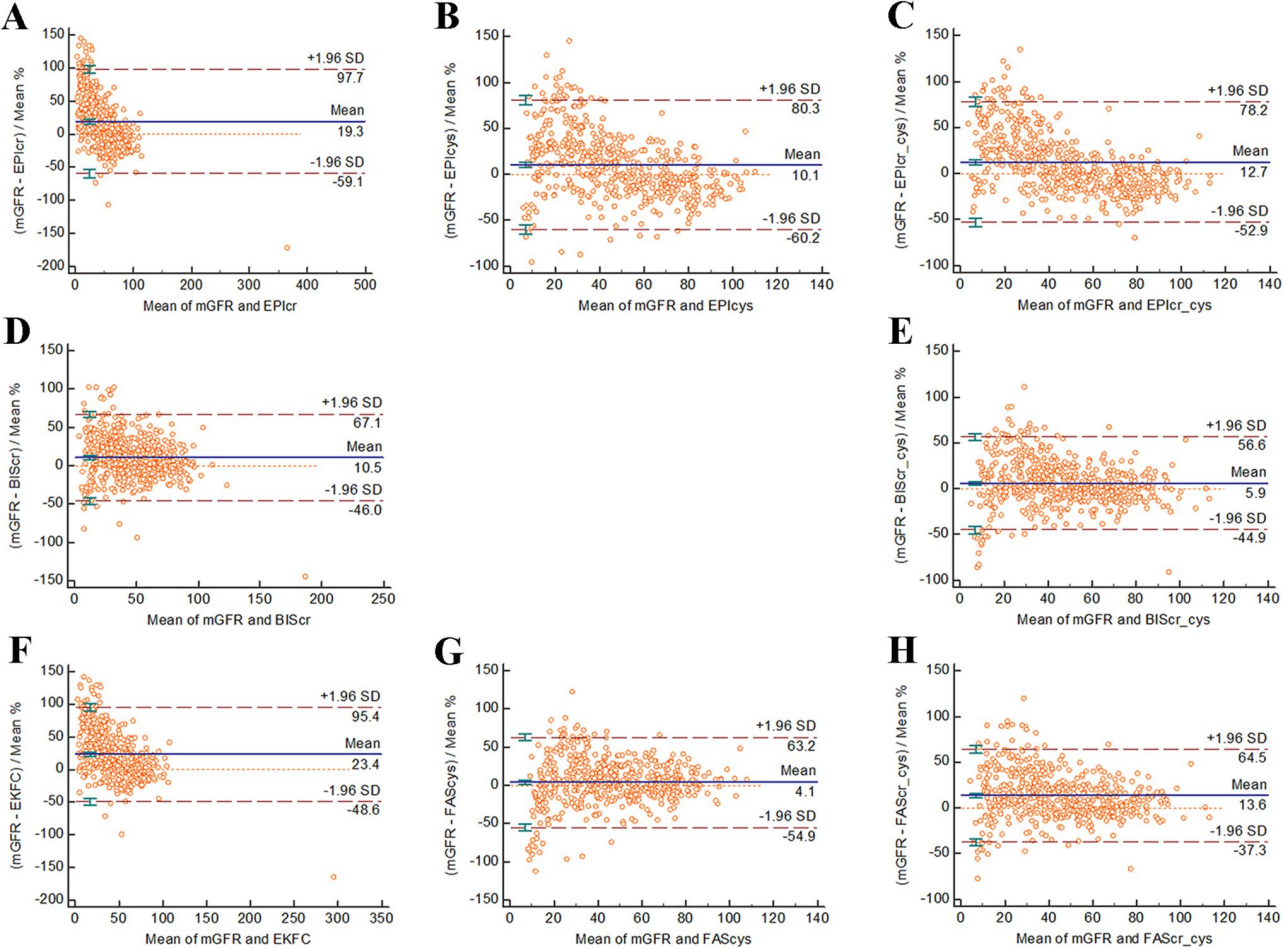
Bland–Altman plots of the 8 equations (**A**–**H**) in older Chinese patients

The mean mGFR and eGFR and the differences between them were plotted on the abscissa and ordinate, respectively. Overall, compared with the other equations, the BIScr-cys and FAScr-cys equations were most consistent with the mGFR, with gaps between the 95% limits of agreement of 101.5 mL/min/1.73 m^2^ and 101.8 mL/min/1.73 m^2^, respectively, followed by the BIScr Eq. (113.1 mL/min/1.73 m^2^) (Fig. 1).

**Fig. 2:**
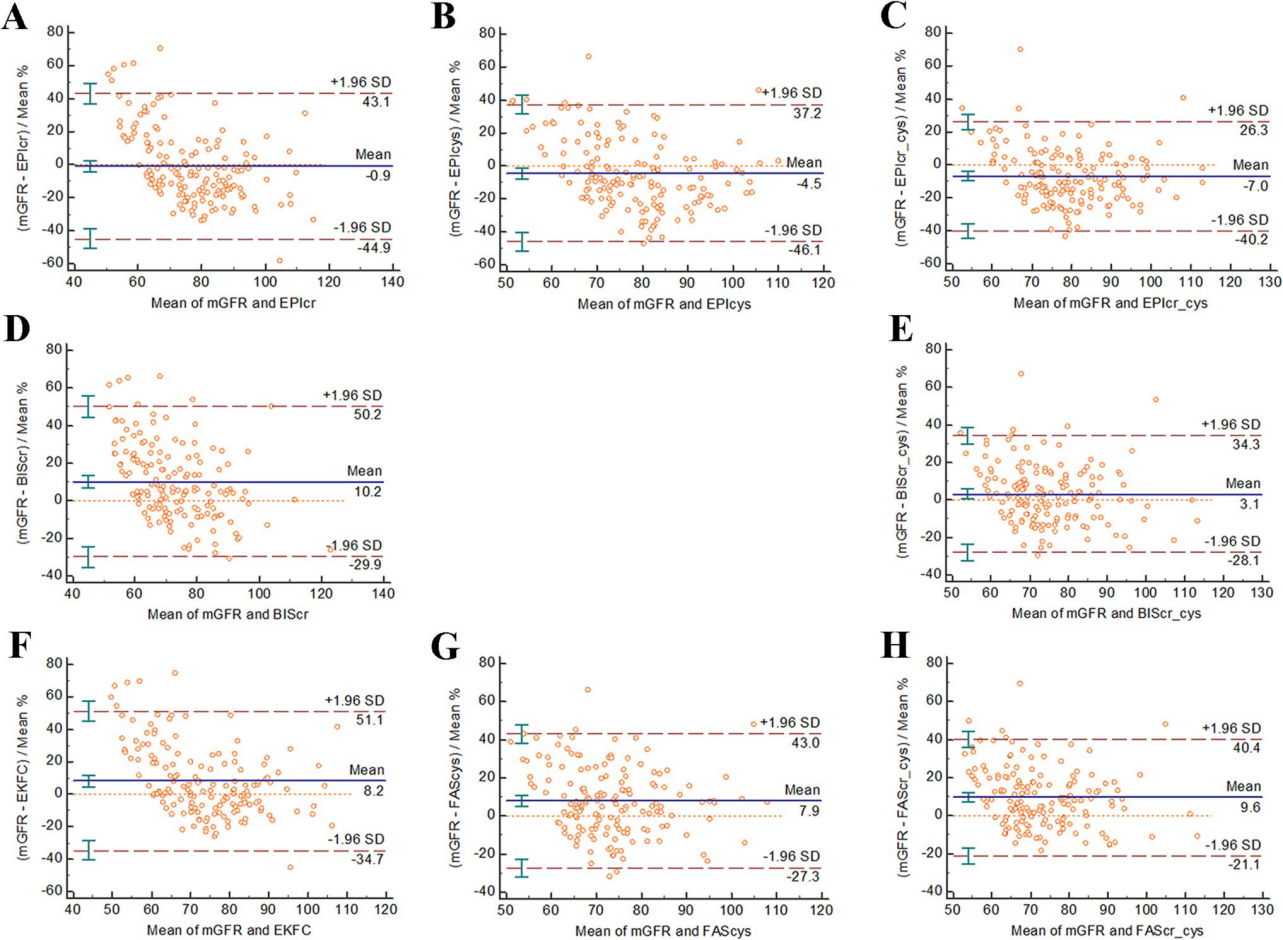
Bland–Altman plots of the 8 equations (**A**–**H**) in older Chinese patients with measured glomerular filtration rate ≥ 60 mL/min/1.73 m^2^

**Fig. 3:**
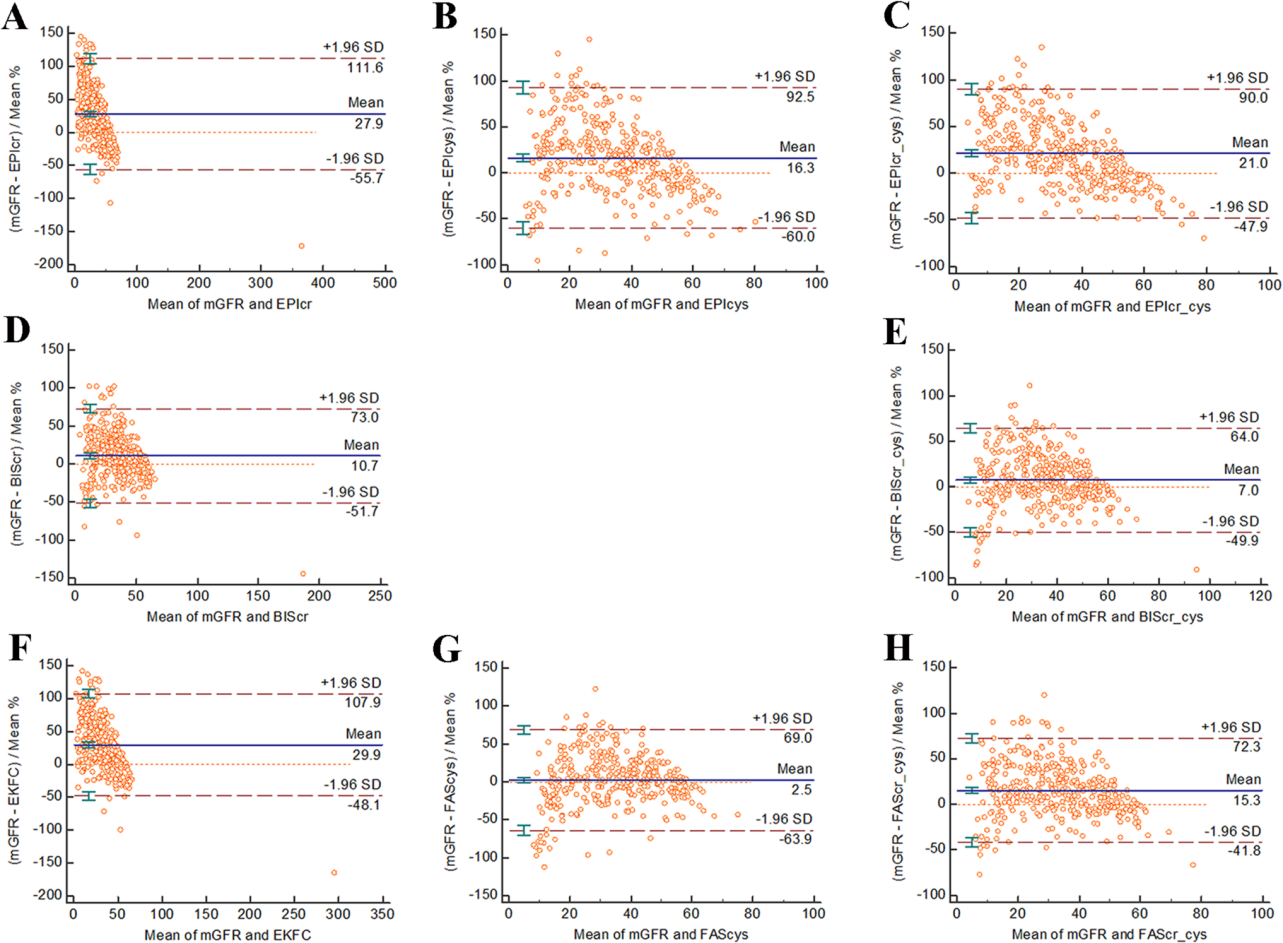
Bland–Altman plots of the 8 equations (**A**–**H**) in older Chinese patients with measured glomerular filtration rate < 60 mL/min/1.73 m^2^

In the mGFR ≥ 60 mL/min/1.73 m^2^ subgroup, the FAScr-cys equation was the most consistent with the mGFR (61.5 mL/min/1.73 m^2^), followed by the BIScr-cys (62.4 mL/min/1.73 m^2^) and CKD-EPIcr-cys (66.5 mL/min/1.73 m^2^) equations (Fig. 2). In the mGFR < 60 mL/min/1.73 m^2^ subgroup, the BIScr-cys equation was the most consistent with the mGFR (113.9 mL/min/1.73 m^2^), followed by the FAScr-cys (114.1 mL/min/1.73 m^2^) and BIScr (124.7 mL/min/1.73 m^2^) equations (Fig. 3).

## Discussion

Of the commonly used eGFR equations, we found that the BIScr-cys was optimal for Chinese patients aged ≥ 65 years, from the perspectives of bias, accuracy, and consistency. Equations based on SCr and SCys performed better in the mGFR ≥ 60 mL/min/1.73 m^2^ subgroup, whereas in the < 60 mL/min/1.73 m^2^ subgroup, although the BIScr-cys was best, neither the accuracy nor consistency was satisfactory.

Although the equations are constantly being updated, accurate GFR assessment in older adults remains difficult. The early CG equations [17] and MDRD equations [18] did not specifically focus on the older. The 2009 CKD-EPI equations are more accurate than the earlier equations but are inappropriate for GFR assessment in older adults [19, 20]. The BIS equations [11] and FAS equations [12] were developed in 2012 and 2016, respectively, and were the first to address the older and all age groups, respectively. Although the BIS and FAS equations evidence less bias and better accuracy compared with earlier equations when used to evaluate the older population [20], no equation is ideal compared to the inulin clearance method [19].

The eGFR equations were derived from studies conducted in European and American populations; Asian, especially East Asians populations require further evaluation. Notably, all equations used in previous studies with relatively large sample sizes were largely based on SCr [19, 20]; SCys was assayed in only a few subjects [21]. SCys is a 13-kDa cysteine proteinase inhibitor produced at a near-constant rate that is freely filtered through the glomerular membrane and then completely reabsorbed (without secretion from proximal tubular cells). SCys is thus an ideal candidate for evaluating renal function [22, 23]. All of the CKD-EPI, BIS, and FAS equations have a form based on both SCr and SCys, and they were shown previously to be superior to those based solely on SCr [10, 11, 24, 25]; our work confirms this. Again, we had SCr, SCys, and 99mTc-DTPA renal dynamic imaging data available from all 519 patients, allowing comprehensive and accurate comparisons among the eGFR equations. This is the key point of our study.

Compared with younger people, the accuracy of GFR estimation in the older is affected more by cancer [25], heart failure [26], diabetes [27], and sarcopenia [28]. Although the CKD-EPI equation is more accurate than previous equations, particularly for the older, [26] and is recommended by the KDIGO [27], this equation was not specifically developed for the older. The 2012 BIS and 2016 FAS equations focus on the older and all ages, respectively. We found that the BIS and FAS equations performed better than did the CKD-EPI equation; it is not surprising that the BIS equation, developed particularly for the older, afforded the best performance. BIS and FAS cohorts included only Caucasians and/or North Africans [11–13]. Ethnic variations may explain the differences between the eGFRs and mGFR. Older patients with a GFR < 60 mL/min/1.73 m^2^ constitute a very special subgroup of patients with stage 3–5 CKD, who are of most concern to clinicians. Unfortunately, no equation performs satisfactorily for this subgroup, not even the BIScr-cys, the accuracy (P30) of which is only 74.73%, thus much lower than that recommended by KDIGO (≥ 90%) [16]. This may be because of the low proportion of older patients with moderate-to-severe renal insufficiency in the cohort used to develop the equation [11].

Renal ECT has been performed in the clinic for decades and standardized procedures have been developed and implemented for many years: in the supine position, 99mTc-DTPA was injected intravenously in a “bullet” fashion, and renal dynamic imaging was performed immediately, with blood flow phase (2 s/frame) to observe the development time of the kidneys and the abdominal aorta, and functional phase (60 s/frame) to observe the uptake and distribution of radioactivity in both kidneys. The 99mTc-DTPA used in this test has a half-life of only 6 h, and the contrast agent used in the test is so small that it is rapidly excreted from the urinary system after entering the bloodstream, so there is very little residue in the body at the end of the test, which is minimally harmful to the human body, and there is a good degree of safety even for multiple tests. It is currently an important method for clinical renal function measurement.

Our work had several limitations. First, 99Tcm-DTPA renal dynamic imaging served as the reference method, thus not a more accurate clearance method compared with using inulin, iohexol, or radioactive 51Cr-EDTA, 99mTc-DTPA, or 125I iothalamate. Also, the patients were relatively small in number and were from a single center; multi-center studies are required to verify our findings.

In conclusion, the BIScr-cys equation is currently the best choice when evaluating older Chinese patients, but no equation is satisfactory when assessing those with stage 3–5 CKD. A new equation for older Chinese or even East Asians is required, especially for those with moderate-to-severe renal insufficiency.

### Supplementary Information

Below is the link to the electronic supplementary material.

Supplementary file1 (DOCX 42 KB)
