# Supplementary material for: Optimal assessment of the glomerular filtration rate in older chinese patients using the equations of the Berlin Initiative Study
Source: Aging Clin Exp Res. 2024 Jan 31;36(1):17. doi: 10.1007/s40520-023-02657-8 (PMC10830815; doi:10.1007/s40520-023-02657-8)
Supplement: Supplementary file 1 — Supplementary file1 (DOCX 42 KB) [file 40520_2023_2657_MOESM1_ESM.docx]

Supplementary Table 1: eGFR equations included in this study.

| CKD-EPI (Chronic Kidney Disease Epidemiology Collaboration) |
| --- |
| CKD-EPIcr:  ${eGFR= 141\times min(\frac{\mathrm{SCr}}{\kappa},1)}^{\alpha}\times{max(\frac{\mathrm{SCr}}{\kappa},1)}^{-1.209}\times{0.993}^{\mathrm{Age}} \left[ \times1.018 if female \right]\left[ \times1.159 if black \right]$, where SCr is serum creatinine, κ is 0.7 for females and 0.9 for males, α is −0.329 for females and −0.411 for males, min is the minimum of SCr/κ or 1, and max is the maximum of SCr/κ or 1.  CKD-EPIcys:  ${eGFR=133\times min(\frac{\mathrm{SCys}}{0.8},1)}^{-0.499}\times{max(\frac{\mathrm{SCys}}{0.8},1)}^{-1.328}\times{0.996}^{\mathrm{Age}}\left[ \times0.932 if female \right]$, where SCys is serum cystatin C, min indicates the minimum of SCr/κ or 1, and max indicates the maximum of SCys/κ or 1.  CKD-EPIcr-cys:  ${eGFR=135\times min(\frac{\mathrm{SCr}}{\kappa},1)}^{\alpha}\times{max(\frac{\mathrm{SCr}}{\kappa},1)}^{-0.601}\times{min(\frac{\mathrm{SCys}}{0.8},1)}^{-0.375}{\times{max(\frac{\mathrm{SCys}}{0.8},1)}^{-0.711}0.996}^{\mathrm{Age}}\left[ \times0.969 if female \right]\left[ \times1.08 if black \right]$, where SCr is serum creatinine, SCys is serum cystatin C, κ is 0.7 for females and 0.9 for males, α is −0.248 for females and −0.207 for males, min indicates the minimum of SCr/κ or 1, and max indicates the maximum of SCr/κ or 1. |
| BIS (Berlin Initiative Study) |
| BIScr:  $eGFR=3736\times{SCr}^{-0.87}\times{Age}^{-0.95}\times0.82 if female$, where SCr is serum creatinine, SCys is serum cystatin C  BIScr-cys:  $eGFR=767\times{CysC}^{-0.61}\times{SCr}^{-0.40}\times{Age}^{-0.57}\times0.87 if female$, where SCr is serum creatinine, SCys is serum cystatin C |
| FAS (full-age-spectrum) |
| EKFC (European Kidney Function Consortium), modified FAScr:  $eGFR=107.3\times{(\frac{\mathrm{SCr}}{Q_{crea}})}^{-0.322}\times{0.990}^{(Age-40)}, if Age >40 and \frac{\mathrm{SCr}}{Q_{crea}}<1$,  $eGFR=107.3\times{(\frac{\mathrm{SCr}}{Q_{crea}})}^{-1.132}\times{0.990}^{(Age-40)}, if Age >40 and\frac{\mathrm{SCr}}{Q_{crea}}\geq1$, where $Q_{crea}$ = 0.90 mg/L for males, and 0.70 mg/L for females.  FAScys:  $eGFR= \frac{107.3}{CysC/Q_{CysC}}\times{0.988}^{(Age-40)}, if Age>40$, where $Q_{CysC}$ = 0.82 mg/L for ages <70 years and $Q_{CysC}$ = 0.95 mg/L for ages ≥70 years,  FAScr-cys:  $eGFR= \frac{107.3}{\alpha\times\frac{SCr}{crea}+(1-\alpha)\times\frac{CysC}{Q_{CysC}}}\times{0.988}^{(Age-40)}, if Age>40$, where α= 0.5，$Q_{CysC}$ = 0.82 mg/L for ages <70 years and $Q_{CysC}$ = 0.95 mg/L for ages ≥70 years |
